# Trends in Patient Complaints in a Pediatric Emergency Department: Comparison of Pre- and Post-Pandemic Periods

**DOI:** 10.1159/pjp/aekag005

**Published:** 2026-10-08

**Authors:** Marlene Marques, Bruna Oliveira Barbosa, Catarina Encarnação, Mafalda Santos, Sofia Reis, Cristina Baptista

**Affiliations:** Department of Pediatrics, Unidade Local de Saúde de Viseu Dão-Lafões, Viseu, Portugal

**Keywords:** Emergency service, Pediatrics, Patient complaints, Waiting time, Quality of health care, Serviço de Urgência Hospitalar, Pediatria, Reclamações de Pacientes, Tempo de Espera, Qualidade da Assistência à Saúde

## Abstract

**Introduction:**

Patient and caregiver satisfaction is a key indicator of health care quality and is particularly relevant in pediatric emergency care. Patient complaints represent an important source of information regarding organizational weaknesses and unmet expectations. The COVID-19 pandemic and recent changes in referral pathways have modified the functioning of these services. This study aimed to analyze patient complaints registered in the pediatric emergency department of a level II hospital between January 2020 and June 2025, characterizing temporal trends, main reasons for dissatisfaction, and patient profiles, and comparing findings with the pre-pandemic period.

**Methods:**

A retrospective, observational, descriptive study based on the analysis of all complaints. Complaints were classified according to the Reader et al. taxonomy, evaluating demographic, clinical, and organizational variables as well as institutional response time. Data from January to June 2025 were available; annual estimates based on linear extrapolation were generated when required for descriptive comparisons.

**Results:**

A total of 166 complaints were analyzed among 153,088 pediatric emergency department visits, corresponding to an overall complaint rate of 0.11%. After reaching their lowest values in 2020 and 2021, complaint rates fluctuated annually, with the highest observed complaint rate recorded during the first semester of 2025. Waiting time remained the main reason for dissatisfaction. An increase in complaints related to staff attitude, hospital logistics, institutional rules, and prior referral processes was observed during the post-pandemic period, including the period following implementation of the “Call Before, Save Lives” project. Most complaints were submitted by mothers and concerned children aged 1–9 years. Institutional response time varied over the study period, with the shortest response times observed during the first semester of 2025.

**Conclusion:**

Patient complaints provide valuable information on persistent structural challenges and evolving patterns of dissatisfaction in pediatric emergency care, representing a useful tool for quality improvement initiatives.

## Resumo

**Introdução:**

A satisfação dos doentes e cuidadores é um indicador central da qualidade dos cuidados de saúde, particularmente relevante na urgência pediátrica. As reclamações constituem uma importante fonte de informação sobre fragilidades organizacionais e expectativas não correspondidas. A pandemia de COVID-19 e alterações recentes nos circuitos de referenciação modificaram o funcionamento destes serviços.

**Objetivo:**

Analisar as reclamações registadas na urgência pediátrica de um hospital de nível II entre janeiro de 2020 e junho de 2025, caracterizando a evolução temporal, principais motivos e perfil dos doentes e cuidadores, comparando com o período pré-pandémico.

**Métodos:**

Estudo observacional retrospetivo e descritivo baseado na análise de todas as reclamações submetidas. As reclamações foram classificadas segundo a taxonomia de Reader et al., avaliando variáveis demográficas, clínicas e organizacionais e o tempo de resposta institucional. Os dados de janeiro a junho de 2025 foram utilizados para gerar estimativas anuais por extrapolação linear, quando necessário para comparações descritivas.

**Resultados:**

Foram analisadas 166 reclamações entre 153.088 episódios de urgência pediátrica, correspondendo a uma taxa global de reclamações de 0.11%. Após atingirem os valores mais baixos em 2020 e 2021, as taxas de reclamação apresentaram flutuações anuais, tendo a taxa de reclamação observada mais elevada sido registada no primeiro semestre de 2025. O tempo de espera manteve-se o principal motivo de insatisfação. Verificou-se aumento de queixas relacionadas com atitude das equipas, logística hospitalar, regras institucionais e referenciação prévia durante o período pós-pandémico, incluindo os anos subsequentes à implementação do projeto “Ligue Antes, Salve Vidas”. A maioria das reclamações foi apresentada por mães e referiu-se a crianças entre 1 e 9 anos. O tempo de resposta institucional variou ao longo do período de estudo, tendo os tempos de resposta mais curtos sido observados no primeiro semestre de 2025.

**Conclusão:**

As reclamações fornecem informação relevante sobre desafios estruturais persistentes e padrões evolutivos de insatisfação na urgência pediátrica, constituindo uma ferramenta útil para a melhoria contínua da qualidade dos cuidados.

## Introduction

Patient and caregiver satisfaction with health care services is one of the essential pillars in the assessment of the quality and effectiveness of care provided, assuming particular relevance in sensitive contexts such as pediatric emergency care, where the needs of children and their families require an effective, empathetic, and specialized approach. Among the various mechanisms for quality assessment, patient complaints stand out by offering a direct perspective on system weaknesses and unmet expectations [1, 2].

Previous studies, including the analysis of complaints registered in the pediatric emergency department between 2016 and 2019 [3], highlighted patterns of dissatisfaction mainly focused on waiting time, communication with the clinical team, and the physical conditions of the facilities.

From 2020 onwards, the COVID-19 pandemic introduced profound changes in the functioning of health care services. The reorganization of hospital pathways, the mandatory use of personal protective equipment, restrictions on the number of accompanying persons, and changes in waiting times and triage priority criteria directly affected patients’ and caregivers’ experience [4, 5]. In addition to organizational changes, the pandemic context contributed to greater emotional vulnerability among caregivers, which may have intensified the negative perception of nonclinical aspects such as accessibility, waiting conditions, or the humanization of care.

In this context, the present study aimed to analyze complaints registered in the pediatric emergency department between January 2020 and June 2025, exploring their frequency, typology, and predominant reasons, and comparing the findings with the pre-pandemic period (2016–2019) to identify temporal trends and potential targets for quality improvement.

## Materials and Methods

### Study Design

A retrospective, observational, descriptive study was conducted based on the analysis of all complaints submitted by patients and their caregivers who attended the pediatric emergency department of a level II hospital between January 2020 and June 2025. A comparative analysis was also performed between the study period (January 2020 to June 2025) and the pre-pandemic period (2016–2019), based on previously collected institutional data. The pre-pandemic data were obtained from a previously published study conducted in the same pediatric emergency department, using the same complaint sources and the same complaint classification system based on the Reader et al. [6] taxonomy. The taxonomy was selected because it provides a structured framework for the analysis of healthcare complaints and had also been applied in the previous analysis of complaints from the same pediatric emergency department (2016–2019), ensuring methodological consistency and enabling direct comparison between the pre-pandemic and post-pandemic periods. All complaints recorded in the Yellow Book, as well as those submitted by email, by post, or through the Directorate-General of Health website, were included. Each complaint was analyzed according to the following criteria:

- reason for complaint (according to the classification described in Table 1);
- identity of the complainant (mother, father, other caregivers, patient, or anonymous);
- patient sex and age group (newborn: 0–28 days; infant: 29 days–12 months; child: 13 months–9 years; adolescent: 10–17 years and 364 days);
- existence of prior referral (primary health care, SNS 24 line – the Portuguese national health telephone triage and advice service, or other hospitals);
- priority level assigned in Manchester Triage (Red, Orange, Yellow, Green, Blue, or White);
- patient destination after medical assessment (discharge, hospitalization, leaving before completion of care, transfer, among others);
- time elapsed between receipt of complaints and issuance of the institutional response (in days).

**Table 1.** Classification of Complaints According to the Taxonomy of Reader et al. [6]

Domain	Category	Subcategory
Relational	Communication and humanity	Attitude of medical staff
		Attitude of nursing staff
		Attitude of other health care professionals
	Patients’ rights	Discrimination among patients
Management	Time and access	Waiting time
		Lack of access to pediatric emergency care/medical specialties
		Referral-related issues (disagreement with the “Call Before, Save Lives” project)
	Institutional and logistical issues	Hospital care rules (requirement to attend the General Emergency Department for assessment by other specialties)
		Internal regulations of the pediatric emergency department
		– Limitation to one accompanying person
		– Access to clinical records
		Hospital logistics (inadequate cleaning, sanitary conditions, lack of food provision)
		Health care workforce recruitment policies
		Costs imposed by the National Health Service
Clinical	Quality and safety	Disagreement with the priority level assigned for medical assessment (Manchester Triage)
		Disagreement with medical evaluation or clinical management

Complaints were classified according to the Reader et al. [6] taxonomy, as described in Table 1. Each complaint was reviewed and classified by one investigator using its categories and subcategories. The investigator was familiar with the taxonomy and applied the same classification criteria used in the previous 2016–2019 analysis. When a complaint included more than one issue, all applicable reasons were recorded. Ambiguous cases were discussed with the research team until consensus was reached. Percentages by reason for complaint were calculated based on the total number of complaints and may exceed 100% due to the presence of multiple reasons in the same complaint. The relative weight of complaints in relation to the total care activity of the pediatric emergency department was also determined, and its temporal evolution was analyzed. Missing or unavailable information was categorized as “Unknown” and retained in the descriptive analysis. Data for 2025 were available only up to June. To facilitate comparison with previous complete years, annual estimates were obtained by linear extrapolation of first-semester data, assuming a similar distribution of complaints and emergency department activity during the second half of the year. These estimates were used for descriptive purposes only and should be interpreted with caution.

### Statistical Analysis

Data were analyzed using Microsoft Excel (Microsoft Corporation, Redmond, WA, USA). No inferential statistical analyses were performed. Descriptive statistics were used to summarize the data. Categorical variables were presented as absolute frequencies and percentages. Continuous variables were described using means and medians, as appropriate. Complaint rates were calculated as the number of complaints divided by the total number of emergency department visits for each year and expressed as percentages. Comparisons between the pre-pandemic (2016–2019) and post-pandemic (2020–June 2025) periods were performed descriptively.

### Ethics

The study was conducted in accordance with the Declaration of Helsinki [7]. This study was reported in accordance with the Strengthening the Reporting of Observational Studies in Epidemiology (STROBE) guidelines [8].

## Results

During the pre-pandemic period (2016–2019), a total of 172 complaints were recorded among 136,114 pediatric emergency department visits, corresponding to an overall complaint rate of 0.13% [3]. Between January 2020 and June 2025, a total of 166 complaints were analyzed among 153,088 pediatric emergency department visits, corresponding to an overall complaint rate of 0.11%. A summary of annual pediatric emergency department activity, patient complaints, complaint rates, waiting-time complaints, complaints with prior referral, and institutional response times is presented in Table 2. The complaint rate showed a dynamic pattern between 2016 and June 2025 (Fig. 1a). During the period 2016–2019, there was a slight downward trend, from 0.13% in 2016 to 0.10% in 2019. This period coincides with the analysis previously published by Meira Nisa et al. [3], which identified a peak in 2017. Between 2020 and 2021, values reached the lowest point of the series (0.08%). Following the lowest complaint rates observed in 2020 and 2021, complaint rates fluctuated annually, reaching an observed complaint rate of 0.18% during the first semester of 2025.

**Table 2.** Annual Pediatric Emergency Department Visits, Complaints, Complaint Rates, Waiting-Time Complaints, Complaints With Prior Referral, and Mean Institutional Response Time, 2020–June 2025

Year	Visits	Complaints	Complaint rate (%)	Waiting-time complaints	Complaints with prior referral	Mean response time (days)
2020	18,356	15	0.08	8	5	18.9
2021	20,532	16	0.08	8	2	17.9
2022	36,149	46	0.13	31	11	11.9
2023	35,086	33	0.09	25	6	16.7
2024	29,204	31	0.11	13	9	19.3
Jun 2025	13,761	25	0.18	15	20	8.5

**Fig. 1.**
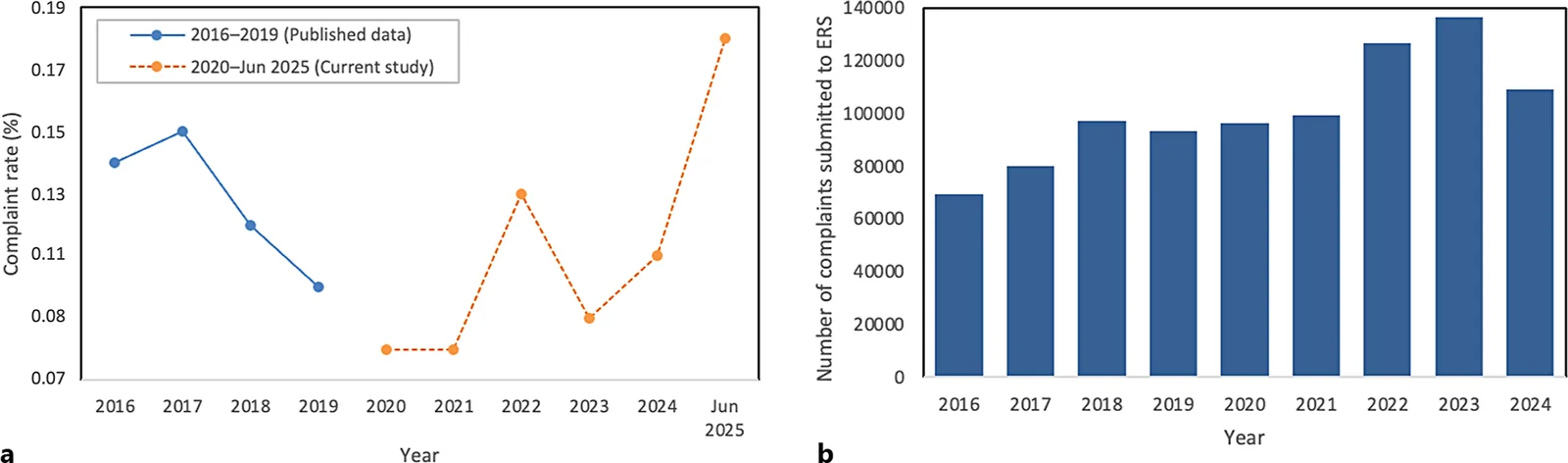
Temporal evolution of patient complaints in the pediatric emergency department: national context. **a** Evolution of the complaint rate in the pediatric emergency department between 2016 and June 2025, expressed as a percentage of total annual emergency department visits; data for 2025 correspond to observed data from January to June 2025. **b** Annual distribution of the number of patient complaints, compliments, and suggestions submitted to the Portuguese Health Regulatory Authority (ERS) between 2016 and 2024.

According to annual reports published by the Portuguese Health Regulatory Authority (ERS) [9], the number of complaints, compliments, and suggestions submitted between 2016 and 2024 increased progressively, reaching a peak in 2023 (136,714 cases) (Fig. 1b). In 2024, a slight decrease was recorded, although values remained above those observed in the pre-pandemic period.

The comparative analysis between the evolution of the complaint rate in the pediatric emergency department (Fig. 1a) and the total volume of cases submitted to ERS (Fig. 1b) reveals that their behavior is not directly proportional over time. While the total number of reports reached a peak in 2023 and declined in 2024, the complaint rate in the pediatric emergency department followed a different pattern, with annual fluctuations and the highest observed complaint rate occurring during the first semester of 2025.

### Characterization of Complaints by Clinical Priority Profile

Patients triaged as “Green” accounted for most complaints in both periods (Fig. 2a). A slight proportional decrease was observed, from 77.3% in 2016–2019 to 74.1% in the period from 2020 to June 2025. There was an increase in the percentage of complaints from the “Orange” (from 1.2% to 3.6%), “White” (from 0.6% to 1.8%), and “Unknown” (from 3.5% to 7.2%) categories.

**Fig. 2.**
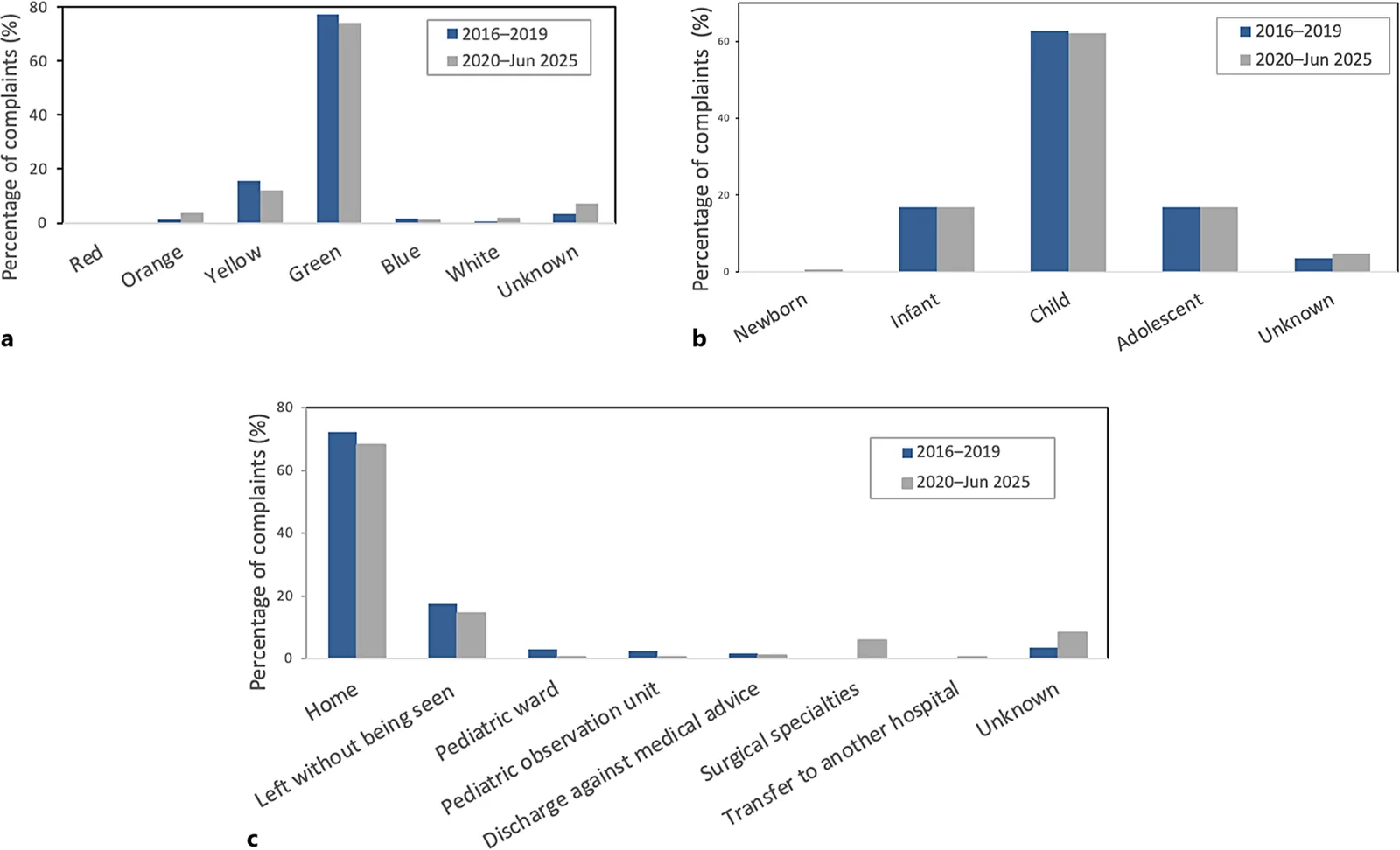
Clinical and demographic characterization of complaints in the pediatric emergency department. **a** Percentage distribution of complaints according to the priority level assigned in Manchester Triage, comparing the periods 2016–2019 and 2020–June 2025. **b** Percentage distribution of complaints according to patients’ age group, comparing the periods 2016–2019 and 2020–June 2025. **c** Percentage distribution of complaints according to patient destination after medical assessment, in the periods 2016–2019 and 2020–June 2025. Percentages were calculated using the total number of complaints in each study period as the denominator.

The age distribution of complaints remained largely stable between the two periods (Fig. 2b). Most complaints referred to children aged 1–9 years, representing 62.8% of complaints in 2016–2019 and 62.0% between 2020 and June 2025. The infant and adolescent age groups maintained similar proportions in both periods, at around 16.9% of complaints. In the more recent period, complaints concerning newborns appeared (0.6%), which had been absent in the previous period. An increase in the “Unknown” category was also observed, from 3.5% to 4.8%.

Most complaints concerned patients discharged home, although their proportion decreased from 72.1% in 2016–2019 to 68.1% in 2020–June 2025 (Fig. 2c). The category “discharge after leaving before completion of care” decreased from 17.4% to 14.5%. A reduction was observed in complaints associated with admission to the pediatric ward and passage through the observation room, both to 0.6%. In the more recent period, new destinations emerged, namely surgical specialties (6.0%) and transfer to another hospital (0.6%). The percentage of complaints with “Unknown” destination increased from 3.5% to 8.4%.

Mothers remained the main authors of complaints (Fig. 3a), increasing from 61.6% to 65.7%. The proportion of complaints made by fathers decreased from 36.1% to 31.3%. Complaints made by the patients themselves and by other caregivers remained residual, representing less than 1% of the total.

**Fig. 3.**
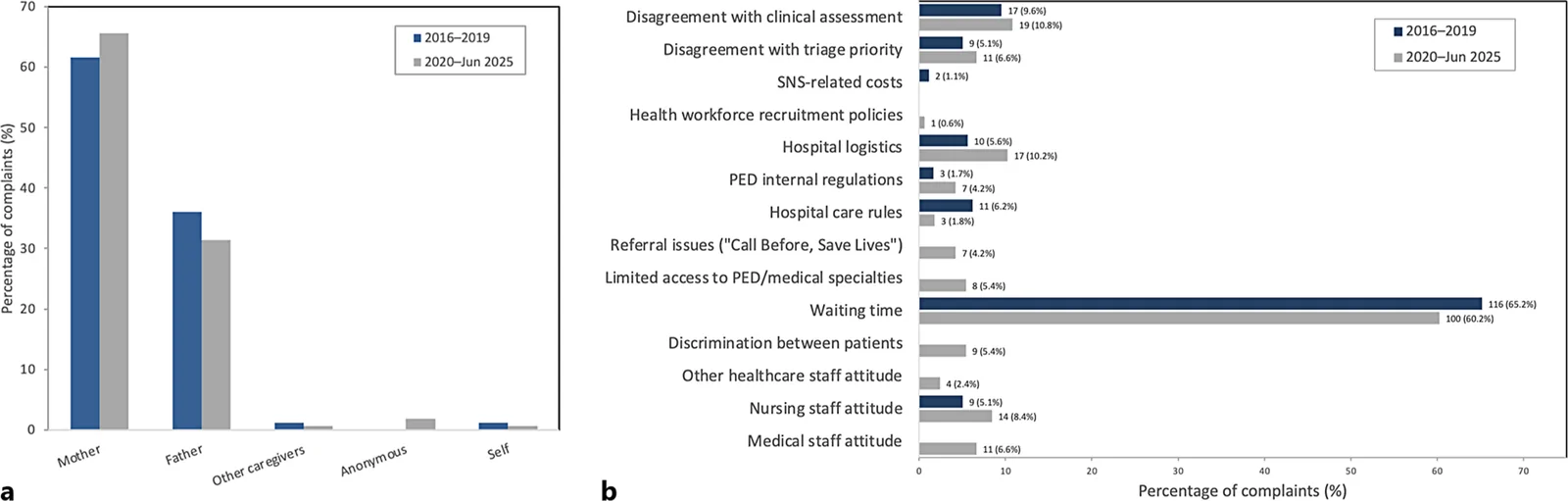
Complainant profile and reasons for dissatisfaction in the pediatric emergency department. **a** Percentage distribution of complainants in the pediatric emergency department according to type of complainant, comparing the periods 2016–2019 and 2020–June 2025. **b** Comparison of the percentage distribution of reasons for complaint by subcategory between the periods 2016–2019 and 2020–June 2025. Percentages were calculated using the total number of complaints in each period as the denominator. Because a single complaint could include more than one reason, the sum of frequencies may exceed the total number of complaints, and the sum of percentages may exceed 100%. PED, pediatric emergency department.

### Reasons for Complaint: Thematic Analysis and Evolution Between Periods

Waiting time remained the leading reason for complaint (Fig. 3b), although with a reduction from 65.2% to 60.2%. Complaints related to nursing staff attitude increased from 5.1% to 8.4%, and in the more recent period complaints also emerged regarding medical staff attitude (6.6%) and other health care professionals (2.4%). Complaints related to hospital logistics increased from 5.6% to 10.2%, and those concerning the internal regulations of the pediatric emergency department rose from 1.7% to 4.2%.

With regard to clinical aspects, dissatisfaction with Manchester Triage increased from 5.1% to 6.6%, and disagreement with the clinical management rose from 9.6% to 10.8%. In the period from 2020 to June 2025, subcategories also emerged relating to discrimination among patients (5.4%), lack of access to medical specialties (5.4%), and problems with referral (4.2%).

### Care Volume, Waiting Times, and Structural Factors of Dissatisfaction

In the aggregated period 2016–2019, 8.7% of complaints corresponded to patients previously referred through institutional pathways (Fig. 4a). From 2020 to 2024, the proportion of complaints involving patients with prior referral fluctuated annually, followed by a marked increase to 80% during the first semester of 2025. Between 2020 and June 2025, the percentage of complaints specifically concerning patients referred by SNS 24 increased from 20% to 72% (Fig. 4b).

**Fig. 4.**
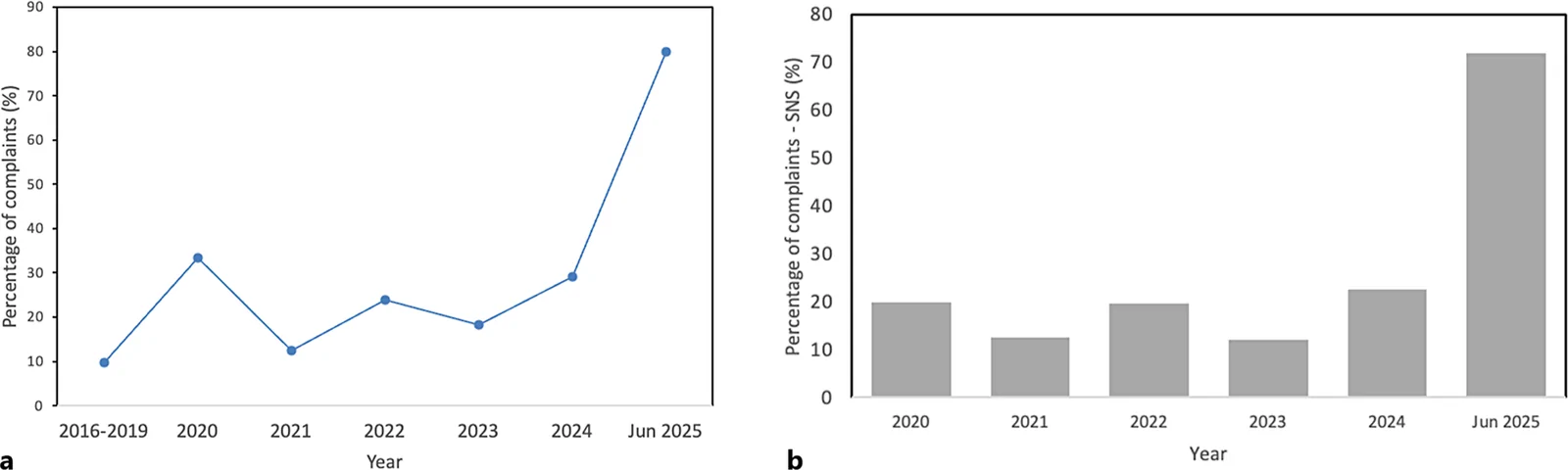
Evolution of prior referral of pediatric emergency department patients. **a** Evolution of the percentage of complaints associated with patients with prior referral to the pediatric emergency department, calculated using the total number of complaints in each period as the denominator, between 2016 and 2019 (aggregated) and 2020–June 2025. Institutional referrals include pathways such as SNS 24, primary health care, other hospitals, or CODU-INEM. **b** Percentage of complaints associated with patients referred via SNS 24, calculated using the total number of complaints in each year as the denominator, between 2020 and June 2025.

Between 2020 and June 2025, most complaints about waiting time were submitted by caregivers of patients triaged as “Green,” ranging from 76.9% to 100%. Mean waiting times in those episodes exceeded the maximum limit of 120 min recommended by Manchester Triage, with values ranging from 178 to 323 min (Fig. 5a).

**Fig. 5.**
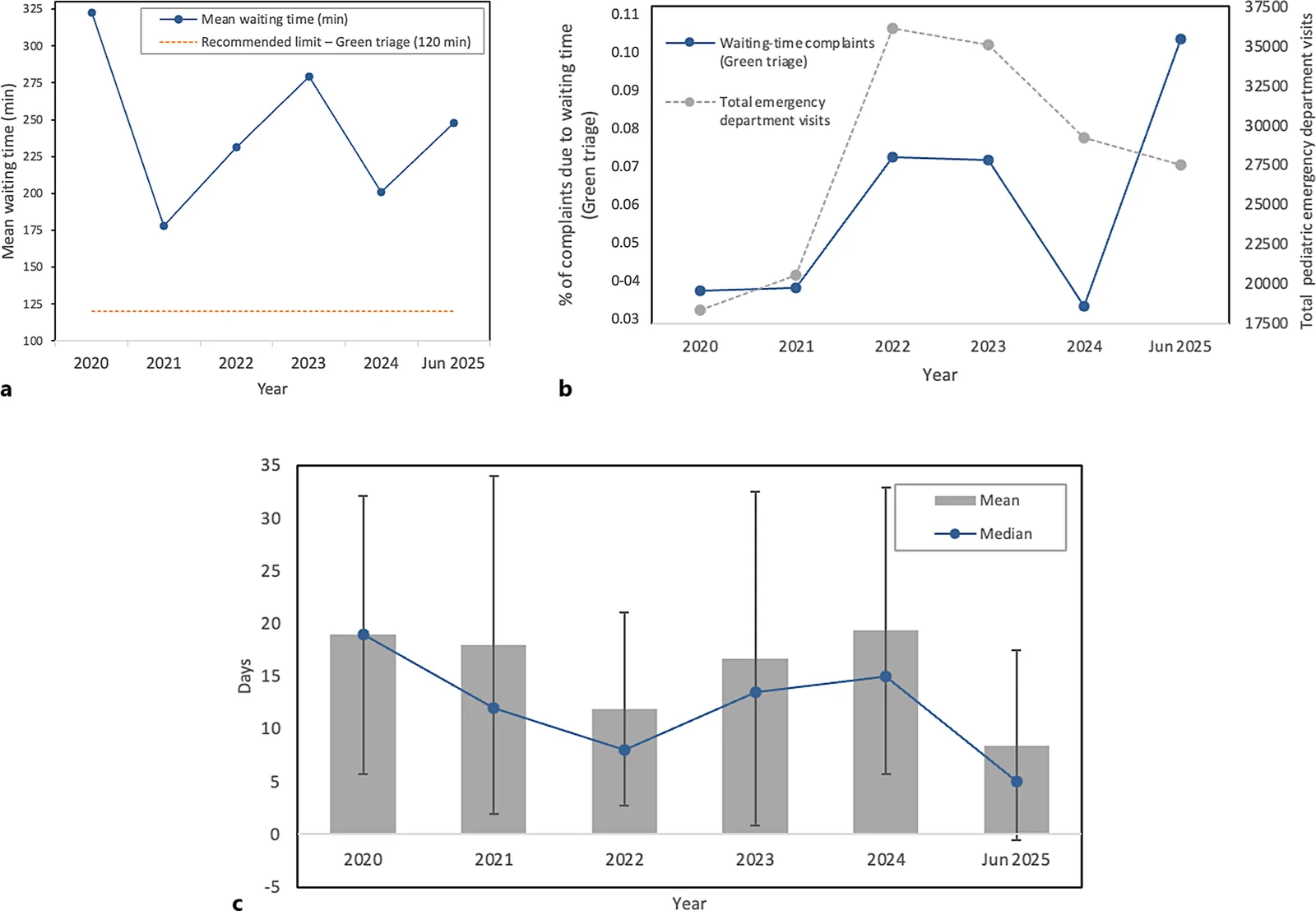
Waiting time and care volume in complaints associated with Green triage in the pediatric emergency department. **a** Annual evolution of mean waiting time (minutes) in episodes with complaints about waiting time among patients triaged as “Green,” between 2020 and June 2025. The dashed line represents the maximum recommended waiting time for “Green” priority according to Manchester Triage (120 min). **b** Evolution of the percentage of waiting-time complaints among patients triaged as “Green,” calculated as the number of waiting-time complaints among “Green” patients divided by the total number of pediatric emergency department visits for that year, between 2020 and June 2025. The percentage for 2025 corresponds to the observed value based on data available up to June. Only the total number of pediatric emergency department visits shown on the secondary axis represents an annual estimate obtained by linear extrapolation. **c** Mean response time to complaints per year in the pediatric emergency department between 2020 and June 2025 (days), with SD shown as error bars.

The evolution of the percentage of waiting-time complaints among patients triaged as “Green,” calculated relative to the total number of pediatric emergency department visits each year, showed a nonlinear pattern between 2020 and June 2025 (Fig. 5b), with lower values in 2020 and 2021 (around 0.038%), followed by an increase in 2022 and 2023, a decrease in 2024, and the highest observed value during the first semester of 2025.

Institutional response time showed temporal variability throughout the study period (Fig. 5c). In 2022, the mean response time was 11.9 days and the median was 8 days. By June 2025, these values had decreased to 8.5 and 5 days, respectively, corresponding to the lowest SD observed during the study period.

## Discussion

To the best of our knowledge, this study is the first post-pandemic longitudinal analysis of patient complaints in a pediatric emergency department in Portugal. The evolution of the complaint rate in the pediatric emergency department shows an initial phase of decline (2016–2019), followed by a minimum in 2020–2021 and annual fluctuations thereafter, with the highest complaint rate observed in the first semester of 2025. The reduction observed in 2020–2021 may be associated with the COVID-19 pandemic [4, 5], namely with service reorganization and lower attendance at emergency departments. The higher complaint rate observed during the first semester of 2025 may reflect greater demand, structural limitations, and increased patients’ and caregivers’ expectations.

National ERS data show an upward trend in the number of submitted cases until 2023, with a slight decrease in 2024 [9]. This pattern may reflect greater awareness among patients and caregivers of their rights, greater accessibility and knowledge of complaint mechanisms, as well as an institutional environment more open to feedback. The divergence between the national curve and the complaint rate in the pediatric emergency department suggests that local evolution may reflect service-specific dynamics rather than a general increase in patient and caregiver engagement in complaint reporting.

As shown in Figure 2a, patients triaged as “Green” represented the majority of complaints in both periods, suggesting persistent dissatisfaction in cases considered less urgent, in line with the previous study. The increasing presence of complaints in less frequent categories such as “Orange” and “White” may reflect higher patients’ and caregivers’ expectations and situations in which expectations are not recognized.

The stability of the age-group distribution suggests that dissatisfaction spans the whole pediatric spectrum, with greater concentration in childhood. The emergence of complaints concerning newborns may reflect greater caregiver sensitivity in this age group. The increase in the “Unknown” category may be related to anonymous complaints or incomplete data.

Waiting time remains the main reason for complaint, despite a slight percentage reduction. This persistence suggests the existence of a structural problem in access and service efficiency. Most complaints about waiting time occur among patients triaged as “Green,” with mean waiting times above the recommended maximum. The mismatch between expected and actual waiting times may compromise patients’ and caregivers’ trust, especially when not adequately contextualized by health care professionals. The absence of clear explanations, associated with possible low health literacy, may increase the negative perception of delay.

Annual variation in waiting times may be related to conjunctural and structural factors. In 2020, the highest value may reflect the direct effects of the first pandemic wave, namely the reorganization of pathways, limitation of human resources, and the need to separate cases. The reduction in 2021 may be related to the population’s fear of attending the emergency department and progressive organizational adaptation. The increase in 2022 and 2023 may reflect the return of previously suppressed demand, staff turnover, and strikes. The variation observed in 2024 may reflect ongoing organizational changes. Although the “Call Before, Save Lives” project was implemented in the institution in October 2024, its late introduction makes it unlikely to have influenced the annual pattern observed that year. One possible explanation for the increase observed in 2025 is the combination of structural and social factors, including increased immigration to Portugal [10], which may have contributed to greater demand for public services and emergency departments by populations facing greater difficulties in accessing primary health care.

The increase in complaints related to staff attitude, hospital logistics, and internal regulations may be associated with emotional overload among teams, more tense working environments, and greater cultural diversity among patients and caregivers, requiring more sensitive communication skills. The increase in complaints regarding triage and clinical management may reflect misalignment between patients’ and caregivers’ expectations and the clinical criteria used, as well as pressure on teams and communication failures regarding therapeutic decisions.

The new subcategories observed (discrimination, lack of access to specialties, and referral problems) may be associated with structural and social changes, inequalities in access, and institutional adjustments in the post-pandemic period. The identified trends are consistent with those reported in other countries, with waiting time and clinical communication frequently indicated as the main reasons for complaint in emergency departments [1, 11]. Evidence from different health systems suggests that waiting time remains a major driver of dissatisfaction in emergency departments, particularly among low-priority cases [11]. The growing adoption of telephone or remote triage systems in different countries has been associated with challenges similar to those observed in this study. In the United Kingdom, the NHS 111 system has been described as an important instrument for the initial guidance of users, but also as a potential source of frustration when there is a discrepancy between the recommendations provided by telephone and the in-person clinical assessment in the hospital setting. In such cases, users often report feeling that their concerns were not valued, which contributes to dissatisfaction and an increase in the number of complaints, despite having followed the recommended institutional pathway [12]. Studies also describe patient dissatisfaction related to cultural discrimination and language barriers, reinforcing the need for humanization strategies, cultural competence, and improved health literacy [13].

The marked increase in complaints associated with prior referral, particularly following implementation of the national “Call Before, Save Lives” project, may reflect organizational changes associated with the new referral pathway. This project, included in Ordinance No. 281/2021 and operationalized in the institution from October 24, 2024, established prior referral for access to the pediatric emergency department through SNS 24, primary health care, private institutions, or CODU-INEM (National Institute of Medical Emergency dispatch and coordination center). The observed evolution may be consistent with greater use of the new referral pathway; however, the increase in complaints among referred patients indicates that prior referral does not eliminate dissatisfaction and may be associated with the experience in the emergency department and with the misalignment between expectations and in-person clinical assessment. The data also suggest that referral-related complaints reflect gaps in institutional communication, low health literacy, and discrepancies between patients’ and caregivers’ perceptions and the clinical criteria adopted.

Finally, the evolution of institutional response time to complaints was characterized by temporal variability, with lower response times observed in 2022 and during the first semester of 2025. The lower response times observed during the most recent study period suggest improvements in the timeliness and consistency of institutional responses to complaints.

## Limitations

This study has several limitations. Its retrospective design depended on the accuracy and completeness of institutional records and may be subject to underreporting, as not all dissatisfied patients or caregivers submit formal complaints. Complaint classification was performed by a single investigator. Although standardized criteria based on the Reader et al. taxonomy were applied and ambiguous cases were discussed with the research team until consensus was reached, no independent second coding or formal assessment of inter-rater agreement was performed, and some degree of subjectivity in assigning complaint categories cannot be excluded. Missing or unavailable information was categorized as “Unknown” and retained in the descriptive analysis. In addition, the relatively small number of complaints per year may have resulted in unstable yearly percentages, and year-to-year variations should therefore be interpreted with caution. The study was based exclusively on descriptive analyses, without inferential statistical testing; therefore, no causal relationships can be established. Furthermore, the detailed waiting-time analysis (Fig. 5) focused exclusively on complaint episodes involving patients triaged as “Green,” as this was the triage category in which waiting-time complaints were most frequently reported. Therefore, these findings should not be interpreted as representing the overall waiting-time performance of the pediatric emergency department. Finally, as data collection ended in June 2025, the findings for 2025 should be interpreted in light of the incomplete annual observation period. The study was conducted in a single institution, which may limit the generalizability of the findings to other pediatric emergency departments.

## Conclusion

The analysis of patient complaints submitted in the pediatric emergency department of a level II hospital between 2020 and June 2025 revealed changes in patterns of patients’ and caregivers’ dissatisfaction over time, which may be associated with pandemic-related factors, institutional changes, and sociodemographic developments. Structural reasons for discontent persist, such as waiting time, especially among patients classified as “Green,” which may reflect persistent organizational challenges.

At the same time, emerging trends can be observed, including an increase in complaints related to prior referral, staff attitude, and interpersonal communication. An increase in complaints involving formal referral pathways such as SNS 24 was observed following implementation of the national “Call Before, Save Lives” project, which was the referral pathway involved in most complaints from referred patients. However, the increase in referrals was not accompanied by a proportional reduction in dissatisfaction, which may indicate unmet expectations or difficulties in the transition between different levels of care.

The growth in complaints related to institutional rules, discrimination, and logistical difficulties also highlights the need for greater sensitivity to recent sociodemographic transformations, such as the increase in the immigrant population and the cultural diversity of patients and caregivers. These changes require adjustments in communication, in the humanization of care, and in the actual accessibility of services.

Finally, institutional response times varied throughout the study period, with lower mean and median response times observed in 2022 and particularly in the first semester of 2025.

In this context, the systematic analysis of complaints emerges as a relevant tool for improving health care quality, allowing anticipation of problems, adjustment of practices, and alignment of services with patients’ and caregivers’ needs. Continuous improvement of pediatric emergency care will depend on the ability to integrate this active listening into a patient-centered model of care that is culturally sensitive, clinically effective, and organizationally sustainable.

## Acknowledgments

The authors used ChatGPT (OpenAI) to support linguistic revision and improve clarity of the manuscript. The scientific content remains the sole responsibility of the authors.

## Statement of Ethics

This study protocol was reviewed and approved by the Ethics Committee of Unidade Local de Saúde de Viseu Dão-Lafões, Approval No. I23570-202506. Written informed consent was waived due to the retrospective nature of the study.

## Conflict of Interest Statement

The authors have no conflicts of interest to declare.

## Funding Sources

This study was not supported by any sponsor or funder.

## Author Contributions

M.M.: study conception, data collection/organization, analysis, manuscript drafting. B.O.B., C.E., M.S., and S.R.: data collection/organization, critical revision of the manuscript. C.B.: supervision, critical revision of the manuscript. All authors approved the final version.

## Data Availability

The data underlying this study are not publicly available because they contain potentially identifiable information derived from institutional complaint records. De-identified or aggregated data may be made available from the corresponding author upon reasonable request, subject to approval by the participating institution and applicable ethical and data protection requirements.
